# Tetradactyl Footprints of an Unknown Affinity Theropod Dinosaur from the Upper Jurassic of Morocco

**DOI:** 10.1371/journal.pone.0026882

**Published:** 2011-12-13

**Authors:** Jaouad Nouri, Ignacio Díaz-Martínez, Félix Pérez-Lorente

**Affiliations:** 1 Faculté des Sciences, Université Mohamed V, Rabat, Morocco; 2 Facultad de Ciencas, Universidad de La Rioja, Logroño, La Rioja, España; 3 Fundación Patrimonio Paleontológico de La Rioja, Enciso, La Rioja, España; Institut de Biologia Evolutiva - Universitat Pompeu Fabra, Spain

## Abstract

**Background:**

New tetradactyl theropod footprints from Upper Jurassic (Oxfordian-Kimmeridgian) have been found in the Iouaridène syncline (Morocco). The tracksites are at several layers in the intermediate lacustrine unit of Iouaridène Formation. The footprints were named informally in previous works “*Eutynichnium atlasipodus*”. We consider as *nomen nudum*.

**Methodology/Principal Findings:**

*Boutakioutichnium atlasicus* ichnogen. et ichnosp. nov. is mainly characterized by the hallux impression. It is long, strong, directed medially or forward, with two digital pads and with the proximal part of the first pad in lateral position. More than 100 footprints in 15 trackways have been studied with these features. The footprints are large, 38–48 cm in length, and 26–31 cm in width.

**Conclusions/Significance:**

*Boutakioutichnium* mainly differs from other ichnotaxa with hallux impression in lacking metatarsal marks and in not being a very deep footprint. The distinct morphology of the hallux of the *Boutakioutichnium* trackmaker –i.e. size and hallux position- are unique in the dinosaur autopodial record to date.

## Introduction

**Figure 1:**
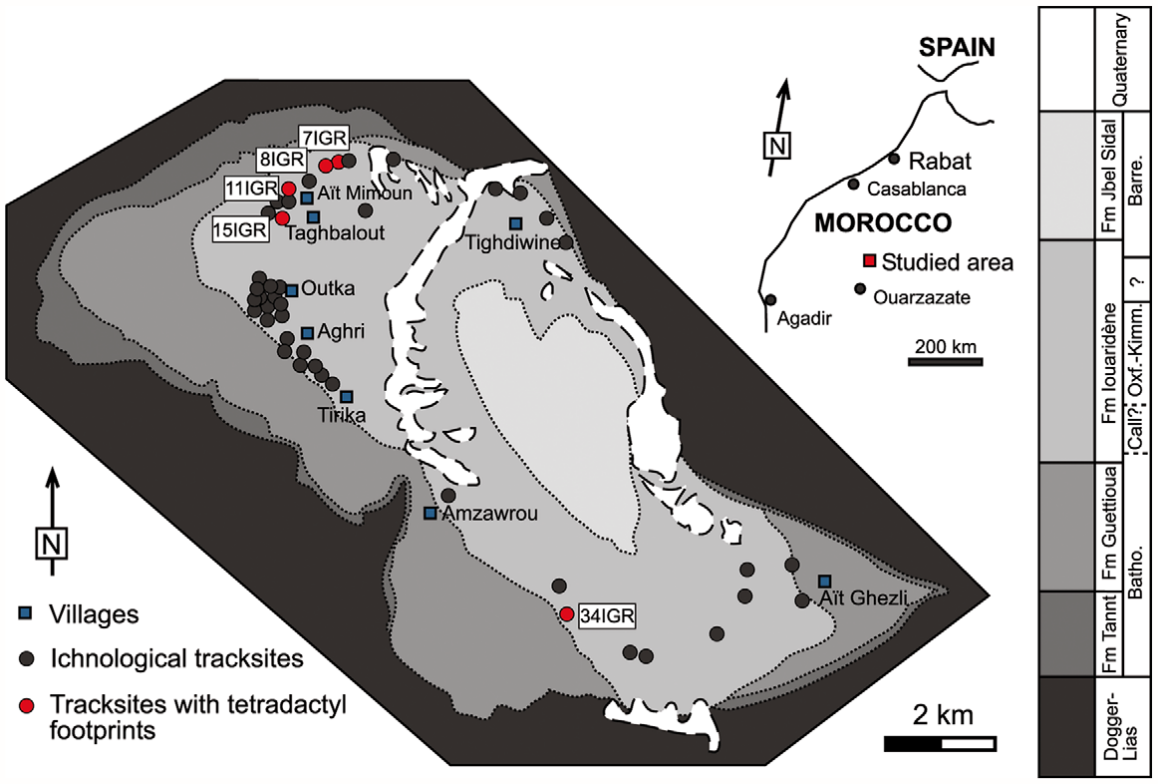
Geologic and geographic location of Iouaridène syncline. Batho-Bathonian; Call.-Callovian; Oxf.-Oxfordian; Kimm.-Kimmeridgian; Barre.-Barremian.

More than 1,500 dinosaur footprints in 43 tracksites (Fig. 1) have been mapped in the research of Iouaridène syncline [1], [2]. According to recent works, the age of the outcrops is Upper Jurassic, Oxfordian-Kimmeridgian [3]. Since the first dinosaur footprints were found in 1937 [1], discoveries and scientific documentation continues.

At the present time, the dating of the 43 cited tracksites [2], new ichnotaxonomic, paleoethologic and paleoecologic contributions is under investigation. The Iouaridene syncline is also noted for its rich ichnodiversity [4]–[8]. Besides sauropod, thyreophoran, and ornitopod footprints [1], there are several theropod ichnotypes [1], [8], [9].

“*Eutynichnium atlasipodus*” [6] was defined in the thesis of Jaouad Nouri as a tetradactyl theropod footprint (I, II, III, IV), with a large and independent hallux with two digital pad impresions [6]. The footprints were included in the icnogenus *Eutynichnium* [10] originally defined in the upper Oxfordian of Cabo Mondego area in Portugal [11]. We consider this ichnotaxon as *nomen dubium* because is defined based on extramorphological features. “*E. atlasipodus*” has not been described formally, thus we consider it *nomen nudum*. The current findings of more footprints with the same characteristics of “*E. atlasipodus*”, and very different of the ichnogenus *Eutynichnium*, suggest the necessity of a formal diagnosis for this type of footprints. The feautures of the hallux of this new ichnotaxon allow the discusión about the position and the shape of digit I (hallux) in theropod dinosaurs.

### Geological setting

The Iouaridéne syncline is located in the Azilal province (Morocco) at East of the High Central Atlas (Fig. 1) in the M'Goun Geopark. The continental “red beds”, are also very common in other basins of the Atlas, in the center of the Iouaridène sinclyne [3], [12]. The red beds are divided into three formations [3]. The lowest, Guetioua Formation, of Bathonian age is composed of red sandstones and claystones, and basic volcanic rocks. The intermediate, Iouaridène Formation, is composed of red detritical rocks from Bathonian?-Callovian to Barremian age. Finally the uppermost, Jbel Sidal Formation, is formed by alternations of medium to coarse sandstones with red claystones of Barremian age.

The Iouaridène Formation is divided into three units [3]. The lower unit is formed maninly by marls and calcretes [12]. The intermediate unit, where the dinosaur footprints have been found, is composed by a superposition of red carbonated shales and red siliceous (silcretes, some with more than 80% SiO_2_) levels with oscillation and current ripples and mud cracks [13]. The upper unit is formed by red sandstones, multicolour shales and thin dolomitic levels [3]. The dolomitic levels of Iouaridène Formation have suggested to some researchers the possiblility of marine environment (carbonate plataform) for these footprints [14]. Recent research indicate a continental origin for all the red beds from the High Atlas [13]. Body fossil remains from the lower and the upper units include vertebrates (principally fishes), charophytes and ostracods. The Iouaridène Formation indicates a lacustrine environment [13].

The age of Iouaridène syncline red beds has been intertpreted to suggest a wide range of: Upper Lias [15]; Bathonian [16]; Bathonian-Callovian [17]; Lower Cretaceous (Infracenomanian) [18]. Currently, the outcrops with dinosaur footprints (the intermediate unit of the Iouaridène Formation) are considered Oxfordian-Kinmeridgian in age, as they lie a few meters below dated Kimmeridgian [3], [12].

### Ichnodiversity and age of Iouaridène syncline footprints

Ichnotaxa from the Iouaridène Formation include: *Megalosaurus* sp. [15], *Eubrontes* ( = *Brontozoum*) ichnosp. [19]; *Breviparopus taghbaloutensis* [20]; *Carmelopodus* ichnosp. [16]; “*Eutynichnium atlasipodus*” [6]; *Kayentapus* ichnosp. [8], [21] y *Megalosauripus* ichnosp. [8], [21]; and *Deltapodus* ichnosp. [22]–[24], wich occur else where in units that have been assigned various Jurassic and Cretaceous ages. Thus, it appears the assemblages is not easily dated on the basis of tracks identifications.

Theropod footprints are the most abundant in the syncline and both small footprints (14 cm) [6] and the largest theropod footprints in the world (90 cm) [9] have been reported. There are booth digitigrade [1], [8], [9] and semiplantigrade tracks [2], [6], [25]. Most of the semiplantigrade footprints (with metatarsal marks) in the Iouaridéne, have also hallux impression [2], [23]. Nevertheless, there are also footprints with an hallux impression without a metatarsal mark. This type of footprints was named “*E. atlasipodus*” [6] and it is restudied herein.

Sauropod footprints are abundant [5], [7], [26]. Ornithopod [6], [25] and thyreophoran [23], [24] footprints have also been reported.

## Materials and Methods

The footprints are designated according to previous convention [1], [2] as follows: first, the tracksite identification; second, the trackway; and third the footprint. For instance, 7IGR6.1 is the first footprint of tracway number 6 of tracksite 7 from IGR (Iguaridene or Iouaridène). To simplify and for consistence, the trackways studied in the Jaouad Nouri thesis with other designations [6], like 1Am8, 8Ta1. etc. have been changed according to previous classification [1], [2]. The equivalences are: 1Am1-8IGR1; 1Am2-8IGR2: 1Am3-8IGR3; 1Am4-8IGR4; 1Am8-8IGR5; 1Ta1-11IGR1; 1Ta2-11IGR2; 2Ta2-11IGR4; 2Ta3-11IGR5; 8Ta1-15IGR5.

The first place where tetradactyl footprints without a metatarsal impresion were found was tracksite 8IGR from Aït Mimoun (8IGR1 and 8IGR3). In subsequent prospectings they were found at the 7IGR, 8IGR, 11IGR, 15IGR and 34IGR tracksites. Trackways 7IGR7, 8IGR1, 8IGR2, 8IGR5, 11IGR1, 11IGR5 and 15IGR5 reveal tetradactyl footprints throughout (75 footprints in total) (see Appendix S1). In other trackways the hallux impression is recognized only in some footprints (7IGR1, 7IGR6, 8IGR3, 8IGR4, 11IGR2, 11IGR4 and 34IGR10).

The measurements (Table 1, Appendix S2, Appendix S3) and nomenclature used in this study are based on other works [27]–[30] principally. Measurements taken were: footprint length (FL), footprint total length -including hallux - (FLt), footprint width (FW), pace length (PL), stride length (SL), trackway deviation (TD), outer trackway width (eTW), pace angulation (ANG), footprint rotation (FR), digit length (I-II-III-IV), digit divarication (I∧II∧III∧IV) and extension of the digit III beyond a line drawn across the tips digit II and IV, measured down the axis of digit III (te). The hip height (H) was estimated with Thulbon [29] formula, and the speed was calculated using the Alexander [31] formula for V1 and the Demathieu [32] formula for V2.

**Table 1:** Means of the trackways with tetradactyl footprints.

	FL	FLt	FW	PL	SL	TD	eTW	ANG	FR	H	I-II-III-IV	I∧II∧III∧IV	V1	V2	te	N°
7IGR1	37	41	24	121	242	4	31	173	1	173	----16--	--13-34	6.5	5.1		14
7IGR6	34	38	26	116	230	4	36	172	-1	161		100-26-39	6.5	5.0	14	22
7IGR7*	38	46	27	114	223	9	44	161	1	174	18-----	49-17-30	5.6	4.7	11	15
8IGR1*	32	38	32	108	213	7	47	164	-1	154	20-18-20-20	55-20-37	6.1	4.8	9.7	5
8IGR2*	39	45	27	131	256	9	45	163	0	181	--26-27-27	44-07-27	6.7	5.3	14	16
8IGR3	38	45	30	120	237	11	53	159	5	177	17-----26	63-16-27	6.1	5.0	13	6
8IGR4	31	36	27	122	241	6	41	168	2	150	13-19---20	66-12-32	7.6	5.1	12	6
8IGR5*	36	43	31	138	267	4	38	172	0.5	168	22-18-23-17	61-11-13	8.1	5.8	12.5	28
11IGR1*	37	43	29	125	250	4	38	175	1	173	20-19-20-22	42-34-32	6.9	5.3	13.2	5
11IGR2	32	37	26	105	208	8	42	167	1	153	19-17-20-19	56-20-24	5.9	4.7	8.8	14
11IGR4	32	41	27	118	230	13	54	154	4	153	16-18-25-20	33-19-22	7.2	5.4	12.2	6
11IGR5*	37	48	29	128	253	8	47	166	5	173	24-18-24-20	22-21-24	7.0	5.4	13.2	6
15IGR5*	31	41	31	111	218	7	37	166	0	151	21-19-23-22	54-16-20	6.5	5.0	12	5
34IGR10	28	40	24	125	261	4	33	173	-3	137	10-16---9	----11-23	9.5	6.2	0.3	4

Abbreviations: see Material and method.

Thulborn [29]: 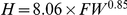


Alexander [31]: 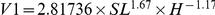


Demathieu [32]: 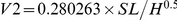


All parameters are given and compared in cm, except ANG, FR and I∧II∧III∧IV in degrees. The parameters have been measured directly in the field or in the laboratory from drawings using AutoCAD software. Subsequently, the measures were observed in the outcrops.

## Results

### Relationship between sedimentary structures and footprints

In this work the study surface where the footprints were registered was examined carefully [33]. The study surface may or may be not the tracking surface (the surface where the dinosaur stepped) [34]. All the surfaces with true footprints in Iouaridène syncline have been found in the hard layers (red siliceous levels) with mud cracks [9]. The undertracks and underprints are in resistant layers with ripples. The number of hard layers varies from the northwest area of the syncline, about 20 layers [22], to the southeast area, where there are places with one hard layer. Currently, in the soft levels (shales) footprints have not been found in the soft levels (shales).

**Figure 2:**
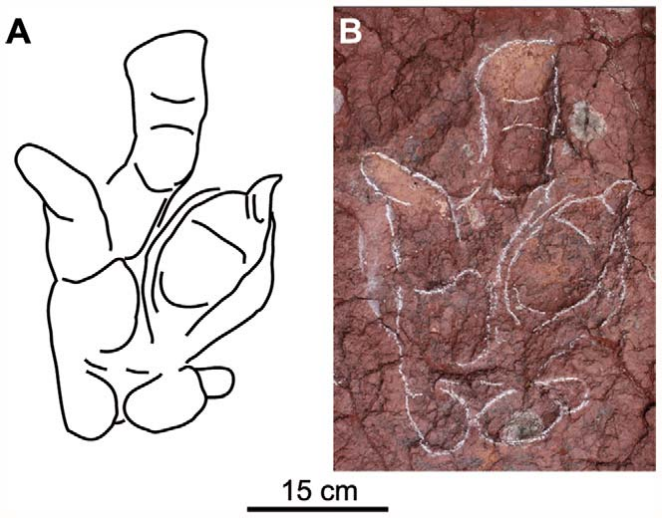
Holotype of *Boutakioutichnium atlasicus*. A) outline. B) photograph.

The footprints studied in this work were registered after the formation of mud cracks. The cracks are deformed by the dinosaur feet so that the sides of the tracks were moved upward and outward (Fig. 2). Sometimes they were also bent, but usually the deformation is closer to an elastic than plastic type. Under the foot, the cracks are broken in small fragments. In the Iouaridène syncline there are also some theropod footprints crossed by mud cracks produced after the dinosaur steps [1]. In the footprint hole, the small rims and the displacement of the mud cracks are due to the dry layer below (elastic or almost) of the tracking surface, were there was a soft zone (of plastic or fluid) mud.

**Figure 3:**
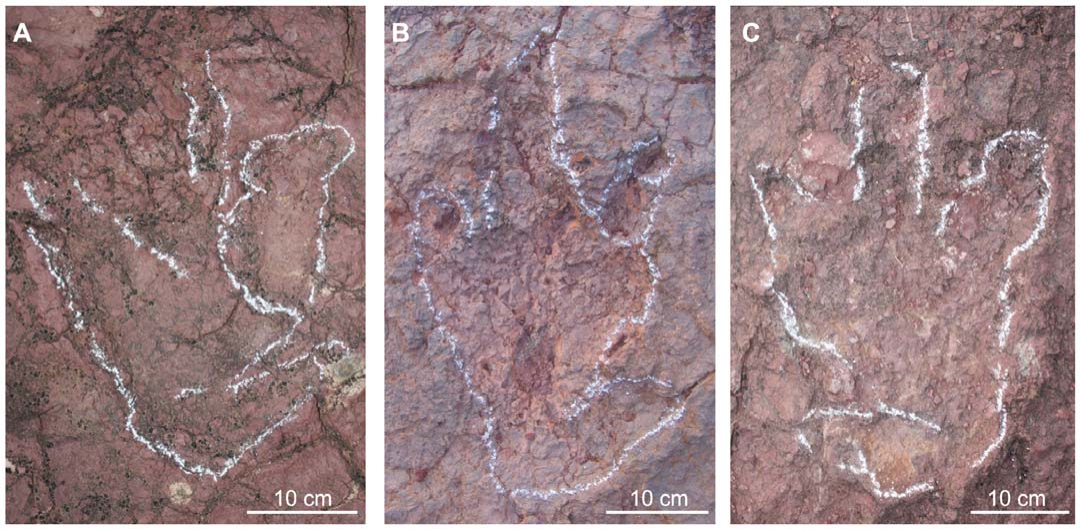
Footprints of *B. atlasicus* from other trackways (see Appendix S1). A) 8IGR1.23. B) 8IGR3.2. C) 8IGR3.3.

In general, the footprint depth is less than 5 cm, therefore the feet do not get any deeper into the mud. Only some footprints (7IGR6.6, 8IGR1.24 footprint, for instance) show collapse structures in the proximal part of the digit III (Fig. 3). This occurs because the mud is accumulated in the interdigital area among the digits.

Most of the footprint shafts have been interpreted as direct structures [35]. Therefore the footprints are considered true footprints and although not all are not an accurate representation of the foot, there are also some elite tracks or stamps . he footprint outline is not always easy to see because sometimes the physical features of the mud cracks do not allow the foot to print it well. The footprint outline does not fit exactly with the foot shape because the mud cracks move as coarse fragments and their behavior is not completely plastic. Nonetheless, in some footprints the diagnostic features as the digits margins, the digital pads and the claws marks are clearly distinghished.

### Nomenclatural acts

The electronic version of this document does not represent a published work according to the International Code of Zoological Nomenclature (ICZN), and hence the nomenclatural acts contained in the electronic version are not available under that Code from the electronic edition. Therefore, a separate edition of this document was produced by a method that assures numerous identical and durable copies, and those copies were simultaneously obtainable (from the publication date noted on the first page of this article) for the purpose of providing a public and permanent scientific record, in accordance with Article 8.1 of the Code. The separate print-only edition is available on request from PLoS by sending a request to PLoS ONE, Public Library of Science, 1160 Battery Street, Suite 100, San Francisco, CA 94111, USA along with a check for $10 (to cover printing and postage) payable to “Public Library of Science”.

In addition, this published work and the nomenclatural acts it contains have been registered in ZooBank , the proposed online registration system for the ICZN. The ZooBank LSIDs (Life Science Identifiers) can be resolved and the associated information viewed through any standard web browser by appending the LSID to the prefix “http://zoobank.org/”. The LSID for this publication is: urn:lsid:zoobank.org:pub: 9383E15A-BC12-404F-B371-32145458FE1B

### Systematic paleoichnology

#### Systematic hierarchy

Dinosauria [36].

Theropoda [37].

### *Boutakioutichnium ichnogen.* nov

ZooBank LSID urn:lsid:zoobank.org:act:3CFC30E8-4E94-4EC8-8448-A9DC9249F3F3.

Etymology. *Boutakioutichnium*, in honor of Dr. Mohamed Boutakiout, professor at the University of Rabat in recognition of his social scientific work and devoted to the protection of M'Goum natural areas (Azilal Province, Morocco), especially its dinosaur footprints outcrops.

Type ichnospecies. *Boutakioutichnium atlasicus.*

### *Boutakioutichnium atlasicus ichnosp.* nov

ZooBank LSID urn:lsid:zoobank.org:act:DA51C3BB-5AA2-4BFB-A5D6-DC15DDA3BA46.

Figure 2, 3. Appendix S1.

#### Synonymy

2007 *Eutynichnium atlasipodus* [6] (*nomen nudum*), p. 113, fig. 115.

2010 “megalosaurian” Morphotype 2D [8], p. 371, fig. 7.

Etymology. *atlasicus*, from Atlas, the name of the mountains where the footprints have been found.

Holotype. Footprint 11IGR1.4 (Fig. 2). It has been deposited a plaster cast in Musée de Géologie d'Azilal, MGP, 1, 2011.7.

Horizon and type locality. Red siliceous levels in the Intermediate unit of Iouaridène.

Formation in the Iouaridène syncline. Upper Jurassic (Oxfordian-Kimmeridgian). Tracksite 11IGR [2], Trackway 11IGR1 [38] near Taghbalout, Azilal province, Morocco. Coordinates UTM 29R698501E3512603N.

Diagnosis: Digitigrade, mesaxonic, tetradactyl (I, II, III, IV) and large theropod footprint of a bipedal dinosaur. All the digits have acuminated ends. Digit I (hallux) has two pads. The hallux is directed proximolaterally or almost perpendicularly to the axis of the foot. The first digital pad of digit I has the proximal area at the same level of the lateral end of digit IV. Digit I (hallux) is almost as long as digit II. Digit III is the longest. Digit II is the widest. There are no metatarsal impressions. Footprint rotation is high. The trackway is very narrow. Dimensions of the holotype are: total footprint length 45 cm (whitout hallux 36 cm); width 30 cm; digits I-II-III-IV length 18-22-26-23 cm; interdigital angles I∧II∧III∧IV 44°-25°-29°.

Description: The height for the hind limb calculated according to Thulborn [29] formula ranges between 150 and 180 cm. The total footprint length, hallux included, ranges between 38 to 48 cm (Table 1, Fig. 3). Whitout hallux, it ranges between 31 to 38 cm. The width shows little variability (between 26 and 31 cm). Digit III is the longest (20–25 cm). Digit IV measures 17 to 32 cm. Digit I (16–24 cm) is usually longer than II (18–19 cm). Divarication angle II∧IV is low (33° to 67°), while I∧II is high and variable (33–100°). I∧III range between 52° and 126°, with lots of data near 80°. Divarication angle II∧III is 10° less than III∧IV. In the good preserved footprints it is possible to distinguish digital pads, even in the digit I (two pads). In other footprints the digital pads are poorly preserved due to the physical characteristics of the mud. All the digits have acuminated end. The pes is relatively narrow according to the (FL-FW)/FW ratio (0.1–0.5). The toe extension (te) of the digit III beyond a line drawn across the tips of digit II and IV is relatively high (12–14 cm).

The trackways are very narrow (TD/WL less than 0.5) with high pace angulation (159–175°) (see Appendix S1). Footprint rotation is low. The relative stride length (Sl/H) indicates that the dinosaur progresses in a walking gait. This data is contrary to the relative high velocity obtained.

The depth has been measured at three points in the good preserved footprints. In the middle of the hallux (0.5–1.8 cm), in the proximal digital pad of digit IV (1.5–2 cm) and in the central pad of digit III (2–3 cm). The distal area of the digits is slightly deeper than the proximal in the footprint soles which not have been eroded. The footprints of the 7IGR tracksite have averages lower than 8IGR tracksite. No criteria have been found to explain the alternation of tetradactyl and tridactyl footprints in some trackways. It is possible that the variation in the depth of the foot sole and the thickness variation of a clay layer are likely causes of this variability.

Digit III proyection (Weems [39] parameter) placed *Boutakioutichnium* close to *Atreipus* [40] and no showing dispersion data (Appendix S4).

## Discussion

### Ichnotaxonomic discussion

The hallux trace is considered as a generic ichnotaxobase. Therefore the footprints of *Boutakioutichnium* are compared with other theropod ichnogenera and ichnospecies with hallux traces described in scientific literature.

Digit I in *Eutynichnium* is slender, associated with the metatarsal impression, and presents two medial digital pads [11], [41]. Nevertheless, *Boutakioutichnium* has a wide digit I, without metatarsal impression and the proximal area of the first pad is lateral. *Bueckeburgichnus* [42] is also different from *Boutakiouthichnium* because has a narrow digit I, sittuated medially and joined with the metatarsal impression [43]. *Picuichnus* [44] is a very well preserved cast. It has metatarsal impression where there is a narrow digit I perpendicular to digit III. The presence of metatarsus, and the hallux shape and disposition, distinguishes it from *Boutakioutichnium*. *Anomoepus isodactylus* [45]–[46] is based on the trackway of a quadruped. The hallux trace is large, with two digital pad impressions directed forwards. It is different from *Boutakioutichnium* because the digit projection is very low, and the first digital pad of digit I is medial to the footprint axis. *Tyrannosauripus* [47] reveal a long digit I without a metatarsal impression. Nevertheless, the digit is narrow and the proximal area of digit I is medial. *Chongqingpus* [48] lacks a metatarsal impression but has residual digit I. *Saurexallopus* [49], like *Boutakioutichnium*, has a digit I with two digital pads [50], [51], but also has thinner digits, greater divarication, and the proximal area of digit I is medial with respect to the footprint axis. *Neoanomoepus* [52] reveal digit I size and digit III projection similar to *Boutakioutichnium*, although it has metatarsal impressions and the proximal area of digit I is medial.

Most of the footprints with hallux are associated with metatarsal impressions [53], tail impressions [54] or they are footprints which penetrate deeply in the mud [55], [56]. In other words, they are either footprints of anomalous gait, or the dinosaur stepped in a soft mud. These latter types of footprints shows gravitational collapse structures in the footprint walls or structures that indicate the penetration of the feet in the mud where the hallux impression appears as a narrow lateral line or grove [55], [57].

According to this discussion, *Boutakioutichnium* is the first difined ichnotaxon that has the proximal area of the first digital pad situated laterally close to the digit IV proximal end, the pads of digit I are as wide as other digits, and digit I is similar o longer than digit II.

### The hallux in theropod dinosaurs

The hallux consists of three bones in the theropod dinosaurs: one metatarsus and two phalanges [58]. Its size and position (relative elevation and divarication) is variable in Theropoda. The metatarsi and the phalanges are reduced (asociated with the cursorial character of the theropods) roughly half of other digits [59]. In many theropod dinosaurs the metatarsi and the phalanges are very small [58]. Nevertheless the therizinosaurids have a long and robust digit I [60].

The hallux varies its position in both relative elevation and divarication respect to the other metatarsi and phalanges [61]. The elevation depends on the metatarsus I length. It is situated in the middle of digit II in some theropods [59]. The proximal area of metatarsus I is separated from the distal one in some dinosaurs [62]. They do not have fixed articulation point, not even a fixed proximal area or a visible fixed point [58].

The divarication depends on the rotation of the metatarsus I. The hallux position of some theropods does not allow a backward orientation (inversion, retroversion) [59]. In the articulated feet, metatarsus I is parallel to metarasus II [59]. Dinosaurs with not reverse hallux have been cited, such as *Coelophysis* [63], *Velociraptor* [64], *Saurornithoides* [64] and *Compsognathus* [65]. Nevertheless, other researchers assert that most of the dinosaurs have the hallux in backward orientation position [29], [66]. Based on the study of theropod footprints with hallux, the theropods should have the digit I orientated backward [29], [55]. But this assertion is valid only for digitigrade footprints. In semiplantigrade footprints, the metatarsus is flat and digit I should be pointed towards the medial or forward. The divarication angle varies from less than 90° to 180°, in birds to 145° [61]. The retroversion is not only characteristic of birds, but the *Scleromochlus* [67] (Triassic) has the same orientation [68]. Hallux orientation is not necessarily a reliable guide to hallux trace orientation. In fact, studies of footprint formation [55] have shown that a posteriorly oriented hallux may in some cases make an anteriorly oriented hallux trace.

### The hallux in Boutakioutichnium

According to the characteristics inferred for *Boutakioutichnium* hallux, digit I of the trackmaker should be long (17–24 cm) and strong, similar to the other digits. The width of the hallux pads are incompatible with a residual metatarsus I. It is almost as long as digit II. Metatarsus I is rotated such that its distal end moves away from the digit II and is placed close to distal area of digit IV. To impress the hallux and not impress the metatarsus, the phalanges would have to had been locate relatively low and parallel to the ground.and the halluxwas directed medially or forward.

Most trackways are composed only by tetradactyl footprints. Nevertheless, there are others with tridactyl footprints too. Three possibilities have been considered taking into account the possibility that the hallux has a higher position than the sole to justify this fact. The first one is that the hallux sole is elevated with respect to the rest of the foot, and the tetradactyl footprints are deeper than those of the tridactyls. The second is the variation of the metatarsus inclination such that the hallux is nearest to the ground depending on the support angle. The last one is the posibility that the hallux is a retractable digit. None of the three hypotesis is justified by the observed data. There is no evidence that the footprints with hallux impression are much deeper than tridactyls. Also not are drag grooves on the proximal area of the footprint showing variation of foot position in the T phase. To justify retractility the metatarsus should be vertical or inclined forward, and this posture is opposite to the movement of limbs.

Based in the deep data of footprint soles (see above) is possible that the variation in the depth of the foot sole and the thickness variation of a clay layer could explain the alternation of tetradactyl and tridactyl footprints in some trackways.

### Trackmaker affinity

The *Boutakioutichnium* trackmaker must have been a biped dinosaur, with a strong foot and digits with acuminated ends. It was a theropod footprint [69]. The digital divarication, the hallux elevation, the lateral position of the proximal area of the hallux are compatible with a theropod trackmaker. There were neoceratosaurs, spinosauroids, megalosaurids, allosaurids, coelurosaurids and tyrannosauroids in the same age as *Boutakioutichnium* (Oxfordian-Kimmeridgian) [58]. Besides, the family Therizinosauroidea appears in the Lower Jurassic [70].

Undoubtedly there are problems concerning the inferred thickness and length that metatarsus I in the *Boutakioutichnium* trackmaker. Almost all the metatarsi I in Theropoda are thin and short [58] and not consistent with strong and long halluxes. Nonetheless, there are long metatarsi I in other theropods [58], [59] without thin limbs like therizinosaurids, that range in age from the Lower Jurassic to the Upper Cretaceous [70]. There are also references to other theropods with funtional and well developed digit I both in the Triassic, *Tawa* [71], and Upper Cretaceous, *Balaur* [72]. However, there are no criteria that show that metatarsi I is rotated. In this work it is assumed that both features (size and position) are those of the *Boutakioutichnium* trackmaker, thus no correlation has been found a between footprints and the autopodial record. It is possible that these footprints are impressed by a theropod whose pes has not been found or by a yet unknown theropod taxon.

### Conclusions

A new theropod ichnotaxon *Boutakioutichnium atlasicus* has been described from the Iouaridène syncline (Morocco). It has been found in several layers in the intermediate unit of Iouaridène Formation of Upper Jurassic (Oxfordian-Kimmeridgian) age. It is mainly characterized by the hallux impression that is unique in the paleoichnological record. It is long, strong, laterally or medially directed, with two digital pads, with the proximal area of the first digital pad in lateral position, and does not have metatarsal impression or sinks deep into the mud.

The position and size of the hallux is also unique compared with the osteological pes record of theropods. Metatarsus I is turned in such away from the distal area of metatarsus II and is placed close to the distal area of metatarsus IV.

## Supporting Information

Appendix S1 **Trackways with all the footprints tetradactyls.**
(TIF)

Appendix S2 **Measurements of the footprints and trackways.** Abbreviations: see Materials and Methods.
(TIF)

Appendix S3 **Tables with data from all trackways.** Abbreviations: see Materials and Methods.
(DOC)

Appendix S4 **Weems parameter.** Abbreviations: see Materials and Methods.
(TIF)
