# Supplementary figures and images for: Tetradactyl Footprints of an Unknown Affinity Theropod Dinosaur from the Upper Jurassic of Morocco
Source: PLoS One. 2011 Dec 13;6(12):e26882. doi: 10.1371/journal.pone.0026882 (PMC3236743; doi:10.1371/journal.pone.0026882)

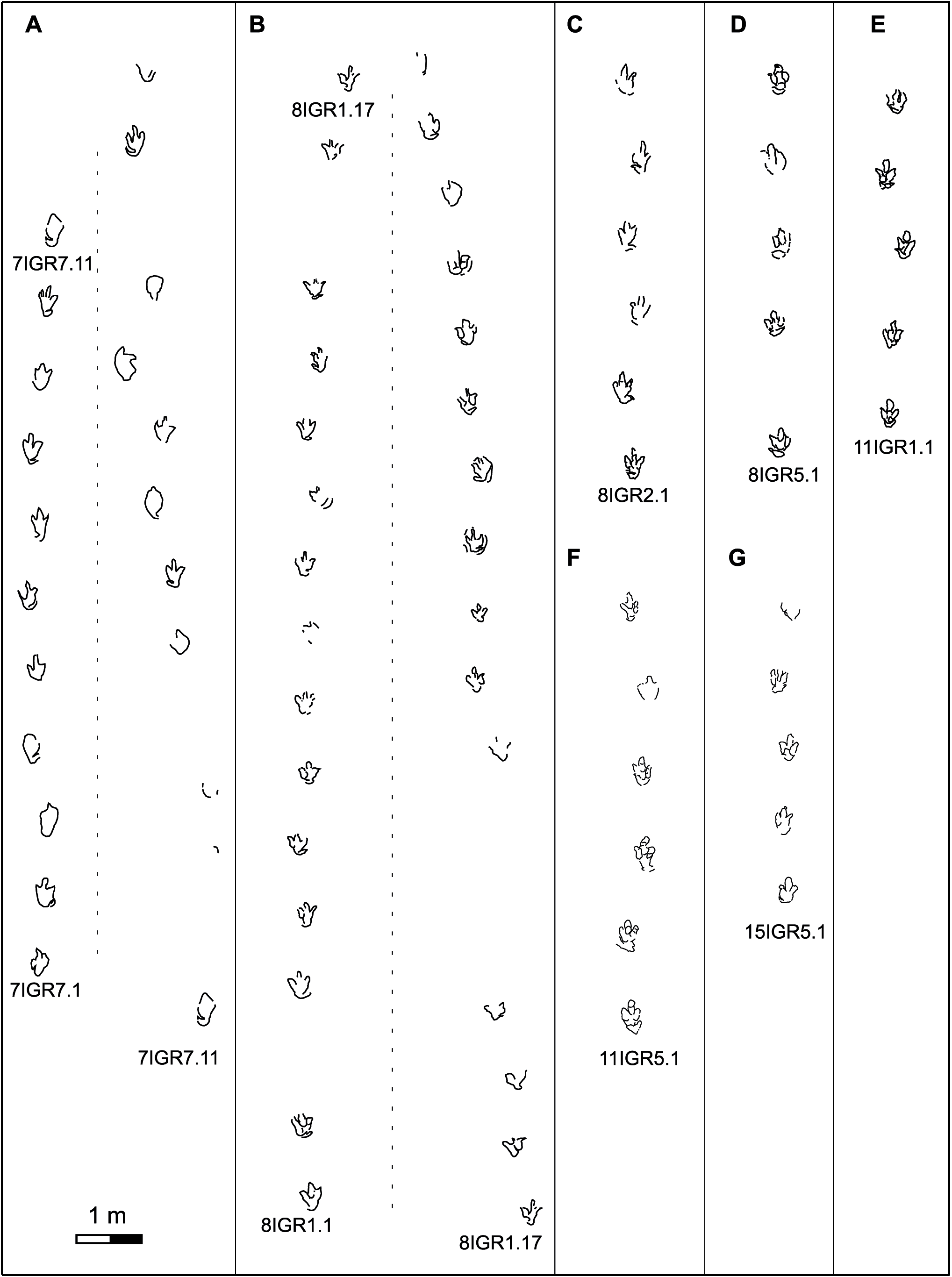

Supplement: Appendix S1 — Trackways with all the footprints tetradactyls. (TIF) [file pone.0026882.s001.tif]

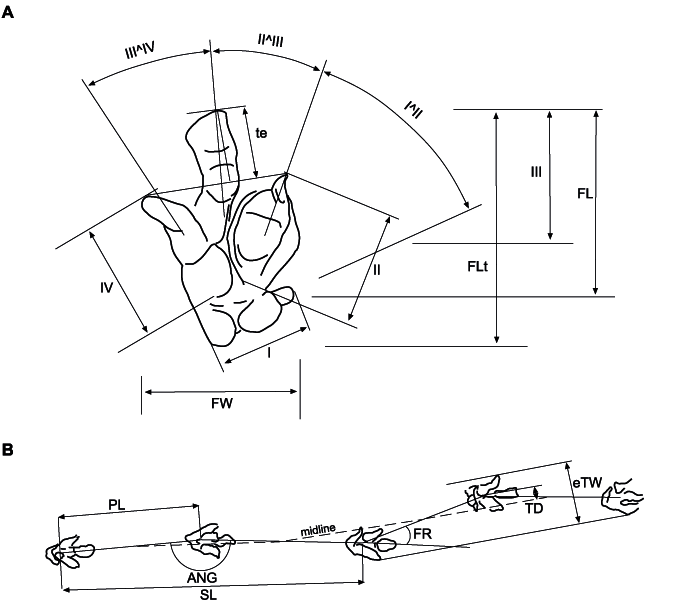

Supplement: Appendix S2 — Measurements of the footprints and trackways. Abbreviations: see Materials and Methods. (TIF) [file pone.0026882.s002.tif]

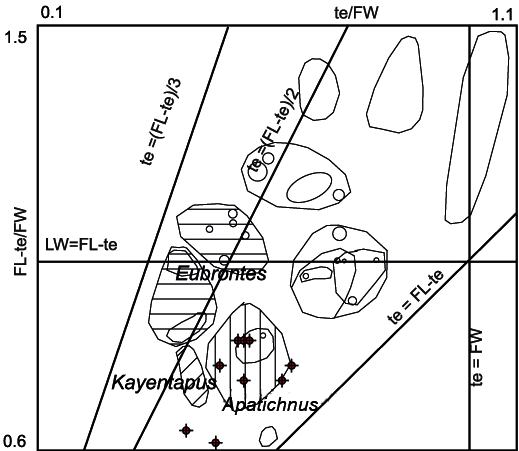

Supplement: Appendix S4 — Weems parameter. Abbreviations: see Materials and Methods. (TIF) [file pone.0026882.s004.tif]
