# Supplementary material for: Tetradactyl Footprints of an Unknown Affinity Theropod Dinosaur from the Upper Jurassic of Morocco
Source: PLoS One. 2011 Dec 13;6(12):e26882. doi: 10.1371/journal.pone.0026882 (PMC3236743; doi:10.1371/journal.pone.0026882)
Supplement: Appendix S3 — Tables with data from all trackways. Abbreviations: see Materials and Methods. (DOC) [file pone.0026882.s003.doc]

|  | FL | FLt | FW | PL | SL | TD | eTW | ANG | FR | H | I-II-III-IV | I^II^III^IV | SL/H | V1 | V2 | (FL-FW)/FW | TD/FW | SL/FL | te |
| --- | --- | --- | --- | --- | --- | --- | --- | --- | --- | --- | --- | --- | --- | --- | --- | --- | --- | --- | --- |
| 7IGR1.20 |  |  | 28 | 122 |  |  |  |  |  |  |  |  |  |  |  |  |  |  |  |
| 7IGR1.19 |  | 38 | 26 | 120 | 245 | 6 | 33 | 169 |  |  |  |  |  | 6.6 | 5.2 |  |  |  |  |
| 7IGR1.18 |  | 44 | 25 | 115 | 238 | 4 | 27 | 172 | -7 |  |  |  |  | 6.3 | 5.0 |  | 0.2 |  |  |
| 7IGR1.17 |  | 46 | 23 | 134 | 249 | 7 | 35 | 168 |  |  |  | ----13-49 |  | 6.8 | 5.3 |  | 0.2 |  |  |
| 7IGR1.16 | 37 | 46 | 30 |  |  |  |  |  |  |  |  |  |  |  |  | 0.2 | 0.3 |  |  |
| ---------- |  |  |  |  |  |  |  |  |  |  |  |  |  |  |  |  |  |  |  |
| ---------- |  |  |  |  |  |  |  |  |  |  |  |  |  |  |  |  |  |  |  |
| ---------- |  |  |  |  |  |  |  |  |  |  |  |  |  |  |  |  |  |  |  |
| ---------- |  |  |  |  |  |  |  |  |  |  |  |  |  |  |  |  |  |  |  |
| ---------- |  |  |  |  |  |  |  |  |  |  |  |  |  |  |  |  |  |  |  |
| ---------- |  |  |  |  |  |  |  |  |  |  |  |  |  |  |  |  |  |  |  |
| 7IGR1.9 |  |  |  | 117 |  |  |  |  |  |  |  |  |  |  |  |  |  |  |  |
| 7IGR1.8 |  | 36 | 22 | 126 | 242 | 6 | 35 | 170 |  |  |  |  |  | 6.5 | 5.1 |  | 0.3 |  |  |
| 7IGR1.7 |  | 40 | 24 | 119 | 244 | 4 | 31 | 175 |  |  |  |  |  | 6.6 | 5.2 |  | 0.2 |  |  |
| 7IGR1.6 |  | 40 | 25 | 114 | 232 | 6 | 33 | 170 | -5 |  |  | -------25 |  | 6.0 | 4.9 |  | 0.2 |  |  |
| 7IGR1.5 |  | 39 | 23 | 122 | 241 | 3 | 28 | 175 | 1 |  |  |  |  | 6.4 | 5.0 |  | 0.1 |  |  |
| 7IGR1.4 |  | 43 | 27 | 122 | 244 | 3 | 29 | 175 | 1 |  |  | -------30 |  | 6.6 | 5.2 |  | 0.1 |  |  |
| 7IGR1.3 |  | 36 | 24 | 118 | 244 | 2 | 31 | 178 | 5 |  |  |  |  | 6.6 | 5.2 |  | 0.1 |  |  |
| 7IGR1.2 |  | 39 | 20 | 126 | 245 | 2 | 30 | 175 | 12 |  |  |  |  | 6.6 | 5.2 |  |  |  |  |
| 7IGR1.1 |  |  |  |  |  |  |  |  |  |  | ----16-- |  |  |  |  |  |  |  |  |
| means | 37 | 41 | 24 | 121 | 242 | 4 | 31 | 173 | 1 | 173 | ----16-- | ----13-34 |  | 6.5 | 5.1 | 0.2 | 0.2 | 6.5 |  |

|  | FL | FLt | FW | PL | SL | TD | eTW | ANG | FR | H | I-II-III-IV | I^II^III^IV | SL/H | V1 | V2 | (FL-FW)/FW | TD/FW | SL/FL | te |
| --- | --- | --- | --- | --- | --- | --- | --- | --- | --- | --- | --- | --- | --- | --- | --- | --- | --- | --- | --- |
| 7IGR6.16 |  |  |  | 131 |  |  |  |  |  |  |  |  |  |  |  |  |  |  |  |
| 7IGR6.15 |  | 34 | 26 | 109 | 239 | 7 |  | 182 | -16 |  |  | ----11-45 | 1.5 | 6.9 | 5.3 | 0.3 |  | 7.0 | 10 |
| 7IGR6.14 |  |  |  | 120 | 227 |  |  | 164 |  |  |  |  | 1.4 | 6.3 | 5.0 |  |  | 6.7 |  |
| 7IGR6.13 |  |  | 27 | 114 | 233 | 6 | 40 | 169 | 3 |  |  | ----34-36 | 1.4 | 6.6 | 5.1 |  | 0.2 | 6.8 | 15 |
| 7IGR6.12 |  | 31 | 24 | 110 | 224 | 3 | 34 | 173 | -1 |  |  | ----21-34 | 1.4 | 6.2 | 4.9 |  | 0.1 | 6.6 | 11 |
| 7IGR6.11 |  | 35 | 24 |  |  |  |  |  |  |  |  | ----30-34 |  |  |  |  |  |  | 13 |
| ---------- |  |  |  |  | 228 |  |  |  |  |  |  |  | 1.4 | 6.4 | 5.0 |  |  | 6.7 |  |
| 7IGR6.9 |  |  | 24 | 124 |  |  |  |  |  |  |  |  |  |  |  |  |  |  |  |
| 7IGR6.8 |  | 39 | 21 | 113 | 237 | 4 | 33 | 173 |  |  |  |  | 1.5 | 6.8 | 5.2 |  | 0.2 | 7.0 | 14 |
| 7IGR6.7 |  |  | 27 | 111 | 224 | 3 | 33 | 174 |  |  |  |  | 1.4 | 6.2 | 4.9 |  | 0.1 | 6.6 | 15 |
| 7IGR6.6 |  | 39 | 25 | 115 | 226 | 2 | 32 | 175 | -3 |  |  | ----17-41 | 1.4 | 6.3 | 5.0 |  | 0.1 | 6.6 | 16 |
| 7IGR6.5 |  | 39 | 27 | 115 | 230 | 4 | 39 | 173 |  |  |  |  | 1.4 | 6.5 | 5.1 |  | 0.1 | 6.8 | 14 |
| 7IGR6.4 | 34 | 39 | 27 | 116 | 230 | 4 | 39 | 172 | 10 | 161 |  | 100-24-38 | 1.4 | 6.5 | 5.1 | 0.3 | 0.1 | 6.8 | 13 |
| 7IGR6.3 |  |  |  | 116 | 231 | 4 |  | 173 | 0 |  |  |  | 1.4 | 6.5 | 5.1 |  |  | 6.8 |  |
| 7IGR6.2 |  | 45 |  | 116 | 232 | 2 | 33 | 175 |  |  |  | ----35-33 | 1.4 | 6.6 | 5.1 |  |  | 6.8 | 18 |
| 7IGR6.1 |  |  | 28 |  |  |  |  |  |  |  |  | ----30-43 |  |  |  |  |  |  | 14 |
| means | 34 | 38 | 26 | 116 | 230 | 4 | 36 | 172 | -1 | 161 |  | 100-26-39 | 1.4 | 6.5 | 5.0 | 0.3 | 0.1 | 6.8 | 14 |

|  | FL | FLt | FW | PL | SL | TD | eTW | ANG | FR | H | I-II-III-IV | I^II^III^IV | SL/H | V1 | V2 | (FL-FW)/FW | TD/FW | SL/FL | te |
| --- | --- | --- | --- | --- | --- | --- | --- | --- | --- | --- | --- | --- | --- | --- | --- | --- | --- | --- | --- |
| 7IGR7.23 |  |  |  | 115 |  |  |  |  |  |  |  |  |  |  |  |  |  |  |  |
| 7IGR7.22 | 36 | 46 | 28 |  |  |  |  |  |  | 169 |  | 56-08-36 |  |  |  | 0.4 |  |  |  |
| 7IGR7.21 |  |  |  | 132 |  |  |  |  |  |  |  |  |  |  |  |  |  |  |  |
| 7IGR7.20 |  |  |  | 116 | 224 | 27 | 83 | 129 |  |  |  |  | 1.3 | 5.6 | 4.7 |  |  | 5.9 | 11 |
| 7IGR7.19 |  |  | 30 | 110 | 214 | 18 | 68 | 143 |  |  |  |  | 1.2 | 5.2 | 4.5 |  | 0.6 | 5.6 |  |
| 7IGR7.18 | 40 | 48 | 27 | 113 | 229 | 11 | 43 | 156 |  | 185 |  |  | 1.3 | 5.9 | 4.9 | 0.5 | 0.4 | 6 |  |
| 7IGR7.17 | 34 | 41 | 28 | 102 | 208 | 5 |  | 169 | 8 | 161 | 18 | 41-18-40 | 1.2 | 5.0 | 4.4 | 0.2 | 0.2 | 5.5 |  |
| 7IGR7.16 | 37 |  |  |  |  |  |  |  |  | 173 |  |  |  |  |  |  |  |  | 12 |
| ---------- |  |  |  |  |  |  |  |  |  |  |  |  |  |  |  |  |  |  |  |
| 7IGR7.14 |  |  |  |  |  |  |  |  |  |  |  |  |  |  |  |  |  |  | 11 |
| 7IGR7.13 |  |  |  |  |  |  |  |  |  |  |  |  |  |  |  |  |  |  |  |
| ---------- |  |  |  |  |  |  |  |  |  |  |  |  |  |  |  |  |  |  |  |
| 7IGR7.11 | 38 | 49 | 26 | 118 |  |  |  |  |  | 177 |  |  |  |  |  | 0.5 |  |  |  |
| 7IGR7.10 | 37 | 47 | 28 | 122 | 224 | 0 | 28 |  | 1 | 173 |  | 52-18---- | 1.3 | 5.6 | 4.7 | 0.3 | 0 | 5.9 | 10 |
| 7IGR7.9 |  | 40 | 25 | 113 | 227 | 1 | 32 | 176 |  |  |  |  | 1.3 | 5.8 | 4.8 |  | 0.0 | 6 | 9 |
| 7IGR7.8 | 40 | 46 | 30 | 115 | 226 | 8 | 40 | 177 | 1 | 185 |  | 48-23-16 | 1.3 | 5.7 | 4.8 | 0.3 | 0.3 | 5.9 | 11 |
| 7IGR7.7 | 40 | 50 | 26 | 111 | 224 | 9 | 42 | 165 | -7 | 185 |  |  | 1.3 | 5.6 | 4.7 | 0.5 | 0.3 | 5.9 | 11 |
| 7IGR7.6 | 37 | 42 | 28 | 112 | 221 | 6 | 49 | 164 | 11 | 173 |  |  | 1.3 | 5.5 | 4.7 | 0.3 | 0.2 | 5.8 |  |
| 7IGR7.5 | 38 |  | 26 | 116 | 227 | 6 | 39 | 166 | -6 | 177 |  |  | 1.3 | 5.8 | 4.8 | 0.5 | 0.2 | 6 | 14 |
| 7IGR7.4 | 34 | 45 | 24 | 119 | 233 | 10 | 45 | 169 |  | 161 |  |  | 1.3 | 6.0 | 4.9 | 0.4 | 0.4 | 6 |  |
| 7IGR7.3 |  | 50 | 28 | 112 | 227 | 9 | 43 | 161 |  |  |  |  | 1.3 | 5.8 | 4.8 |  | 0.3 | 6 |  |
| 7IGR7.2 |  | 44 | 28 | 107 | 220 | 0 | 26 | 163 |  |  |  |  | 1.3 | 5.5 | 4.7 |  | 0 | 5.8 | 11 |
| 7IGR7.1 |  | 41 | 26 |  |  |  |  |  |  |  |  |  |  |  |  |  |  |  | 12 |
| means | 38 | 46 | 27 | 114 | 223 | 9 | 44 | 161 | 1 | 174 | 18 | 49-17-30 | 1.3 | 5.6 | 4.7 | 0.4 | 0.2 | 5.9 | 11 |

|  | FL | FLt | FW | PL | SL | TD | eTW | ANG | FR | H | I-II-III-IV | I^II^III^IV | SL/H | V1 | V2 | (FL-FW)/FW | TD/FW | SL/FL | te |
| --- | --- | --- | --- | --- | --- | --- | --- | --- | --- | --- | --- | --- | --- | --- | --- | --- | --- | --- | --- |
| 8IGR1.34 |  |  |  |  |  |  |  |  |  |  |  |  |  |  |  |  |  |  |  |
| 8IGR1.33 | 29 | 41 | 32 | 102 |  | 5 | 41 | 170 | 3 | 140 | 12------ | ----14-21 |  |  |  | -0.1 | 0.1 |  |  |
| 8IGR1.32 |  |  | 32 | 106 | 210 | 8 | 49 | 163 |  |  |  |  |  |  |  |  | 0.2 |  |  |
| 8IGR1.31 |  |  | 32 | 107 | 210 | 4 |  | 174 |  |  | 11-22----- |  |  |  |  |  | 0.1 |  |  |
| 8IGR1.30 | 35 | 40 |  | 112 | 217 | 3 |  | 174 | -3 | 165 | 16-17----- | 63-17--- | 1.3 | 5.7 | 4.7 |  |  | 6.2 |  |
| 8IGR1.29 | 35 | 44 | 31 | 106 | 217 | 6 |  | 167 | 3 | 165 | 22-17-24-24 | 41-13-25 | 1.3 | 5.7 | 4.7 | 0.1 | 0.2 | 6.2 | 10 |
| 8IGR1.28 | 31 | 38 | 32 | 104 | 207 | 9 | 49 | 160 | -5 | 150 | 22-19-21-- | 67-10-24 | 1.4 | 5.9 | 4.7 | -0.0 | 0.3 | 6.6 | 8.7 |
| 8IGR1.27 | 31 | 37 | 30 | 109 | 210 | 4 | 40 | 168 | 10 | 150 | 21-17----- | 68-17-37 | 1.4 | 6.0 | 4.8 | 0.0 | 0.1 | 6.7 | 10 |
| 8IGR1.26 |  |  | 30 | 106 | 214 | 4 | 37 | 178 | -1 |  | 16-16---17 | 46-31-41 |  |  |  |  | 0.1 |  |  |
| 8IGR1.25 |  | 36 | 30 | 105 | 211 | 11 | 52 | 157 | 7 |  | --16---19 | ----07-40 |  |  |  |  | 0.3 |  | 11.2 |
| 8IGR1.24 | 35 | 37 | 31 | 116 | 216 |  |  |  |  | 165 | 19-17-19-- | 43-12-38 | 1.3 | 5.7 | 4.7 | 0.1 |  | 6.2 | 16.2 |
| 8IGR1.23 |  |  |  |  |  |  |  |  |  |  |  |  |  |  |  |  |  |  |  |
| 8IGR1.22 |  |  |  |  |  |  |  |  |  |  |  |  |  |  |  |  |  |  |  |
| 8IGR1.21 | 26 | 32 | 32 |  |  |  |  |  |  | 130 | 23------ | 79-28-36 |  |  |  | -0.2 |  |  | 8.7 |
| 8IGR1.20 | 39 | 43 | 39 | 115 |  | 7 | 55 | 163 | -4 | 180 | --15----- | ----19--- |  |  |  | 0 | 0.2 |  |  |
| 8IGR1.19 | 29 | 36 | 32 | 100 | 212 | 6 | 50 | 165 | 4 | 140 | 16-15-19-22 | 56-19-45 | 1.5 | 6.7 | 5.0 | -0.1 | 0.2 | 7.4 | 10 |
| 8IGR1.18 | 31 | 39 | 34 | 106 | 205 | 14 | 59 | 150 | – | 150 | 25-16---- | 43-------- | 1.4 | 5.8 | 4.7 | -0.1 | 0.4 | 6.6 |  |
| 8IGR1.17 |  |  | 35 | 110 | 234 |  |  |  |  |  | 20-18---- | 57-14-49 |  |  |  |  |  |  |  |
| 8IGR1.16 |  |  |  |  |  |  |  |  |  |  |  |  |  |  |  |  |  |  |  |
| 8IGR1.15 | 22 | 32 | 32 |  | 214 |  |  |  |  | 114 | --22---- | ----17-50 | 1. 9 | 8.6 | 5.6 | -0.3 |  | 9.5 | 4.4 |
| 8IGR1.14 | 34 | 40 | 34 | 112 |  | 8 | 45 | 163 |  | 163 | 25-17---- | 47-------- |  |  |  | 0.0 | 0.2 |  | 3.7 |
| 8IGR1.13 |  |  | 32 | 108 | 217 | 13 |  | 156 | 0 |  |  | 63-17-33 |  |  |  |  | 0.4 |  |  |
| 8IGR1.12 | 30 | 35 |  | 100 | 204 | 10 |  | 157 | -10 | 145 | 16---17- - - | 34-35---- | 1.4 | 6.0 | 4.7 |  |  | 6.8 |  |
| 8IGR1.11 | 30 | 35 |  | 109 | 204 | 9 |  | 160 | 5 | 145 | 19-16---- | 79-19---- | 1.4 | 6.0 | 4.7 |  |  | 6.8 |  |
| 8IGR1.10 |  |  |  | 106 | 211 | 6 |  | 161 |  |  |  |  |  |  |  |  |  |  |  |
| 8IGR1.9 |  |  | 31 | 108 | 212 | 7 |  | 167 | 0 |  | --- - - - -16 | 70-25-49 |  |  |  |  | 0.24 |  |  |
| 8IGR1.8 | 29 | 37 | 32 | 106 | 212 | 6 | 41 | 167 | -9 | 140 | 22-18-14- - | 56-22-31 | 1.5 | 6.7 | 5.0 | -0.1 | 0.2 | 7.4 |  |
| 8IGR1.7 | 39 | 44 | 30 | 115 | 219 | 8 | 45 | 163 | 6 | 180 | 23- -29- - - - | 60-13-19 | 1.2 | 5.2 | 4.6 | 0.3 | 0.3 | 5.6 | 15 |
| 8IGR1.6 | 36 | 40 | 31 | 109 | 221 | 6 | 42 | 167 | -20 | 170 | 24-19- - - - - | 44-20---- | 1.3 | 5.7 | 4.7 | 0.2 | 0.2 | 6.1 |  |
| 8IGR1.5 | 34 | 40 | 35 | 111 | 219 |  |  |  |  | 160 | 26-22- - - - - | 43-31-52 | 1.4 | 6.0 | 4.8 | -0.0 |  | 6.5 | 9.4 |
| 8IGR1.4 |  |  |  |  |  |  |  |  |  |  |  |  |  |  |  |  |  |  |  |
| 8IGR1.3 |  |  |  |  | 210 |  |  |  |  |  | 19 - - - - - - - | 55------- |  |  |  |  |  |  |  |
| 8IGR1.2 | 35 | 41 | 32 | 109 |  |  |  |  |  | 165 | --16 - - - - - | 43-35-45 |  |  |  | 0.1 |  |  | 8.7 |
| 8IGR1.1 |  |  |  |  |  |  |  |  |  |  |  |  |  |  |  |  |  |  |  |
| means | 32 | 38 | 32 | 108 | 213 | 7 | 47 | 164 | -1 | 154 | 20-18-20-20 | 55-20-37 | 1.4 | 6.1 | 4.8 | -0.0 | 0.2 | 6.8 | 9. 7 |

|  | FL | FLt | FW | PL | SL | TD | eTW | ANG | FR | H | I-II-III-IV | I^II^III^IV | SL/H | V1 | V2 | (FL-FW)/FW | TD/FW | SL/FL | te |
| --- | --- | --- | --- | --- | --- | --- | --- | --- | --- | --- | --- | --- | --- | --- | --- | --- | --- | --- | --- |
| 8IGR2.6 |  |  |  | 137 |  |  |  |  |  |  |  |  |  |  |  |  |  |  |  |
| 8IGR2.5 |  | 45 | 26 | 124 | 258 | 10 | 48 | 162 | 7 |  | ---26---29 | -----05-24 | 1.4 | 6.8 | 5.4 |  | 0.4 | 6.6 | 14 |
| 8IGR2.4 |  | 46 | 30 | 127 | 249 | 5 | 38 | 170 | -9 |  | -----29-- | -----03-22 | 1.4 | 6.5 | 5.3 |  | 0.2 | 6.4 | 14 |
| 8IGR2.3 | 39 | 46 | 25 | 131 | 254 | 10 | 47 | 162 | 2 | 181 | -------26 | 44-10-23 | 1.4 | 6.7 | 5.3 | 0.56 | 0.4 | 6.5 | 13 |
| 8IGR2.2 |  | 39 | 25 | 135 | 262 | 12 | 49 | 159 | -2 |  |  | ----07-33 | 1.4 | 7.0 | 5.4 |  | 0.5 | 6.7 | 14 |
| 8IGR2.1 |  | 41 | 29 |  |  |  |  |  |  |  | ---- 25-- | ----13-32 |  |  |  |  |  |  | 15 |
| means | 39 | 45 | 27 | 131 | 256 | 9 | 45 | 163 | 0 | 181 | -- 26- 27-27 | 44-07-27 | 1.4 | 6.7 | 5.3 | 0.56 | 0.4 | 6.5 | 14 |

|  | FL | FLt | FW | PL | SL | TD | eTW | ANG | FR | H | I-II-III-IV | I^II^III^IV | SL/H | V1 | V2 | (FL-FW)/FW | TD/FW | SL/FL | te |
| --- | --- | --- | --- | --- | --- | --- | --- | --- | --- | --- | --- | --- | --- | --- | --- | --- | --- | --- | --- |
| 8IGR3.6 |  | 46 |  | 118 |  |  |  |  |  |  |  |  |  |  |  |  |  |  |  |
| 8IGR3.5 |  |  |  | 121 | 232 | 11 |  | 158 | 2 |  | 16---------- | 69-19-27 | 1.3 | 5.9 | 4.9 |  |  | 6.1 |  |
| 8IGR3.4 | 35 | 44 | 30 | 116 | 235 | 10 | 54 | 161 | 8 | 165 |  | 54-17-22 | 1.3 | 6.0 | 4.9 | 0.17 | 0.3 | 6.2 |  |
| 8IGR3.3 |  |  |  | 126 | 236 | 12 | 54 | 159 | 6 |  | 21--------25 | 73-10-10 | 1.3 | 6.1 | 5.0 |  |  | 6.2 |  |
| 8IGR3.2 | 41 | 47 | 30 | 120 | 244 | 11 | 52 | 159 | 4 | 189 | 14--------28 | 56-18-38 | 1.4 | 6.4 | 5.1 | 0.37 | 0.4 | 6.4 | 13 |
| 8IGR3.1 | 37 | 46 | 29 |  |  |  |  |  |  |  |  |  |  |  |  |  |  |  | 13 |
| means | 38 | 45 | 30 | 120 | 237 | 11 | 53 | 159 | 5 | 177 | 17--------26 | 63-16-27 | .3 | 6.1 | 5.0 | 0.27 | 0.3 | 6.2 | 13 |

|  | FL | FLt | FW | PL | SL | TD | eTW | ANG | FR | H | I-II-III-IV | I^II^III^IV | SL/H | V1 | V2 | (FL-FW)/FW | TD/FW | SL/FL | te |
| --- | --- | --- | --- | --- | --- | --- | --- | --- | --- | --- | --- | --- | --- | --- | --- | --- | --- | --- | --- |
| 8IGR4.16 |  |  |  | 116 |  |  |  |  |  |  |  |  |  |  |  |  |  |  |  |
| 8IGR4.15 |  | 42 | 27 | 151 | 267 | 4 | 33 | 174 |  |  |  | -----17----- | 1.8 | 9.0 | 6.1 |  | 0.1 | 12.7 |  |
| 8IGR4.14 | 21 | 25 | 20 | 128 | 280 | 5 | 35 | 171 | 10 | 107 |  | 70--22--33 | 1.9 | 9.8 | 6.4 | 0.05 | 0.2 | 13.3 |  |
| 8IGR4.13 |  | 37 | 26 | 132 | 249 | 1 | 28 | 177 | -4 |  | 11-13---13 | ----07-34 | 1.7 | 8.0 | 5.7 |  | 0.0 | 12 |  |
| 8IGR4.12 |  | 37 | 24 | 116 | 236 | 11 | 50 | 159 | 6 |  |  | ----06-44 | 1.6 | 7.3 | 5.4 |  | 0.5 | 11.2 | 13 |
| 8IGR4.11 | 31 |  | 28 | 114 | 232 | 11 | 54 | 159 | 20 | 149 | ---19----- | ----17-26 | 1.5 | 7.1 | 5.3 | 0.11 | 0.4 | 11.0 | 13 |
| 8IGR4.10 | 32 | 38 | 31 | 125 | 240 | 5 | 43 | 170 | -2 | 153 | --18-26-21 | ----14-38 | 1.6 | 7.6 | 5.5 | 0.03 | 0.2 | 11.4 | 10 |
| 8IGR4.9 |  | 37 | 26 | 117 | 237 | 11 | 55 | 159 | 5 |  | ----19------- | ----09-13 | 1.6 | 7.4 | 5.4 | 0.03 | 0.0 | 11.3 |  |
| 8IGR4.8 | 30 | 38 | 29 | 118 | 228 | 1 | 52 | 159 | -3 | 145 | 12--------19 | 56-02-37 | 1.5 | 6.9 | 5.2 | 0.04 | 0.4 | 10.8 | 11 |
| 8IGR4.7 | 29 | 35 | 28 | 128 | 236 | 2 | 32 | 177 | -2 | 141 | ---17-----17 | ----19-25 | 1.6 | 7.3 | 5.4 | 0.37 | 0.1 | 11.2 | 11 |
| 8IGR4.6 | 37 |  | 27 | 111 | 232 | 3 | 33 | 175 | 0 | 173 | ---20-----24 | ----15-36 | 1.5 | 7.1 | 5.3 | 0.10 | 0.1 | 11.0 | 13 |
| 8IGR4.5 | 32 | 36 | 29 | 127 | 238 | 5 | 38 | 171 | 5 | 153 | 17-16----19 | 76-10-34 | 1.6 | 7.0 | 5.4 |  | 0.2 | 11.3 | 11 |
| 8IGR4.4 |  | 38 | 26 | 146 | 271 | 7 | 44 | 168 | 10 |  | ----27------ |  | 1.8 | 9.2 | 6.2 |  | 0.3 | 12.9 |  |
| 8IGR4.3 |  | 38 | 28 | 85 | 230 | 6 | 41 | 168 | -3 | 177 | -----------27 | --------34 | 1.5 | 7.0 | 5.3 | 0.36 | 0.2 | 11.0 | 12 |
| 8IGR4.2 | 32 | 38 | 31 | 113 | 199 | 6 | 43 | 167 | -10 | 153 | ----20----19 | ----08-32 | 1.3 | 5.5 | 4.5 | 0.03 | 0.2 | 9.5 | 11 |
| 8IGR4.1 |  |  |  |  |  |  |  |  |  |  |  |  |  |  |  |  |  |  |  |
| means | 31 | 36 | 27 | 122 | 241 | 6 | 41 | 168 | 2 | 150 | 13-19----20 | 66-12-32 | 1.6 | 7.6 | 5.1 | 0.14 | 0.2 | 10.8 | 12 |

|  | FL | FLt | FW | PL | SL | TD | eTW | ANG | FR | H | I-II-III-IV | I^II^III^IV | SL/H | V1 | V2 | (FL-FW)/FW | TD/FW | SL/FL | te |
| --- | --- | --- | --- | --- | --- | --- | --- | --- | --- | --- | --- | --- | --- | --- | --- | --- | --- | --- | --- |
| 8GR5.5 | 38 | 45 | 32 |  |  |  |  |  |  | 177 | 20-19--15 | 67-07-15 |  |  |  | 0.2 |  |  | 13 |
| 8GR5.4 |  |  |  | 123 |  | 3 |  | 173 |  |  |  |  |  |  |  |  |  |  |  |
| 8GR5.3 | 39 | 47 | 30 | 127 | 248 | 5 |  | 170 | -12 | 181 | 21-18-26-18 | 69-06-06 | 1. 4 | 6.4 | 5.2 | 0.3 | 0.2 | 6.4 |  |
| 8GR5.2 | 30 | 38 | 30 | 122 | 247 | 3 | 38 | 173 | 13 | 145 | 23------ | 56-20-20 | 1.7 | 8.3 | 5.8 | 0 | 0.1 | 8.2 |  |
| 8GR5.1 | 36 | 44 | 32 | 182 | 304 |  |  |  |  | 169 | 25--20-17 | 53-13-12 | 1.8 | 9.7 | 6.5 | 0.1 |  | 8.4 | 12 |
| means | 36 | 43 | 31 | 138 | 267 | 4 | 38 | 172 | 0.5 | 168 | 22-18-23-17 | 61-11-13 | 1.6 | 8.1 | 5.8 | 0.1 | 0.1 | 7.7 | 12.5 |

|  | FL | FLt | FW | PL | SL | TD | eTW | ANG | FR | H | I-II-III-IV | I^II^III^IV | SL/H | V1 | V2 | (FL-FW)/FW | TD/FW | SL/FL | te |
| --- | --- | --- | --- | --- | --- | --- | --- | --- | --- | --- | --- | --- | --- | --- | --- | --- | --- | --- | --- |
| 11IGR1.5 |  |  |  |  |  |  |  |  |  |  | 18------ |  |  |  |  |  |  |  |  |
| 11IGR1.4 | 38 | 45 | 30 | 115 |  | 4 | 40 | 171 | 0 | 177 | 21-21-20-24 | 44-27-36 |  |  |  | 0.3 | 0.1 |  | 14 |
| 11IGR1.3 | 36 | 43 | 28 | 120 | 232 | 4 | 40 | 178 | 3 | 169 | 23-19-16-24 | 45-34-27 | 1.4 | 6.2 | 5.0 | 0.3 | 0.1 | 6.5 | 12.5 |
| 11IGR1.2 | 37 | 42 | 29 | 137 | 255 | 3 | 34 | 175 | -3 | 173 | 19-16--19 | 40-31-39 | 1. 5 | 7.0 | 5.4 | 0.3 | 0.1 | 6. 9 | 13.5 |
| 11IGR1.1 | 37 | 42 | 29 | 127 | 263 |  |  |  | 0 | 173 | ------- | ----------- | 1.5 | 7.4 | 5.6 | 0.3 |  | 7.1 | 13 |
| means | 37 | 43 | 29 | 125 | 250 | 4 | 38 | 175 | 1 | 173 | 20-19-20-22 | 42-34-32 | 1.4 | 6.9 | 5.3 | 0.3 | 0.1 | 6.8 | 13.2 |

|  | FL | FLt | FW | PL | SL | TD | eTW | ANG | FR | H | I-II-III-IV | I^II^III^IV | SL/H | V1 | V2 | (FL-FW)/FW | TD/FW | SL/FL | te |
| --- | --- | --- | --- | --- | --- | --- | --- | --- | --- | --- | --- | --- | --- | --- | --- | --- | --- | --- | --- |
| 11IGR2.12 | 33 |  | 24 |  |  |  |  |  |  | 157 | --20-21-22 | ----04-09 |  |  |  | 0.4 |  |  | 7 |
| 11IGR2.11 |  |  | 27 | 104 |  | 5 | 33 | 170 | 0 | – | --11-21-21 | ----18-18 |  |  |  |  | 0.2 |  | 10 |
| 11IGR2.10 | 30 |  | 25 | 104 | 207 | 2 | 31 | 175 | 3 | 147 | ----19-- | ----21-30 | 1.4 | 6.0 | 4.8 | 0.2 | 0.1 | 6.8 |  |
| 11IGR2.9 |  |  |  | 100 | 203 | 7 |  | 166 |  |  |  |  |  |  |  |  |  |  |  |
| 11IGR2.8 | 30 | 35 |  | 110 | 208 | 5 |  | 170 | -15 | 145 | --20-20-20 | 56-21-28 | 1.4 | 6.2 | 4.8 |  |  | 6.9 | 10 |
| 11IGR2.7 | 32 |  | 27 | 123 | 232 | 24 | 72 | 180 | 0 | 153 | ----16-- | ----32-34 | 1.5 | 7.0 | 5.2 | 0.2 | 0. 9 | 7.2 | 8 |
| 11IGR2.6 | 32 |  | 26 | 105 | 205 | 10 | 45 | 159 | 14 | 153 | ----20-16 | ----24-21 |  | 5.7 | 4.6 | 0.2 | 0.4 |  | 7 |
| 11IGR2.5 | 31 | 38 | 28 | 105 | 205 | 2 | 30 | 176 | 5 | 149 | 20-17-21-18 | ----12-25 |  | 5.9 | 4.8 | 0.1 | 0.1 |  | 5.5 |
| 11IGR2.4 |  |  | 26 | 100 | 204 | 8 | 42 | 161 | -5 |  | --18-23-- | ----26-26 |  |  |  |  |  |  |  |
| 11IGR2.3 | 32 | 39 | 25 | 104 | 200 | 9 | 41 | 161 | 8 | 153 | 19--22-20 | ----17-26 |  | 5.5 | 4.5 |  |  |  |  |
| 11IGR2.2 | 34 |  | 27 | 101 | 202 |  |  |  |  | 161 | --16-21-19 | ----23-17 | 1.2 | 5.2 | 4.4 | 0.3 |  | 5.9 | 11 |
| 11IGR2.1 |  |  |  |  |  |  |  |  |  |  |  |  |  |  |  |  |  |  |  |
| means | 32 | 37 | 26 | 105 | 208 | 8 | 42 | 167 | 1 | 153 | 19-17-20-19 | 56-20-24 | 1.4 | 5.9 | 4.7 | 0.2 | 0.3 | 6.7 | 8.8 |

|  | FL | FLt | FW | PL | SL | TD | eTW | ANG | FR | H | I-II-III-IV | I^II^III^IV | SL/H | V1 | V2 | (FL-FW)/FW | TD/FW | SL/FL | te |
| --- | --- | --- | --- | --- | --- | --- | --- | --- | --- | --- | --- | --- | --- | --- | --- | --- | --- | --- | --- |
| 11IGR4.14 |  |  |  |  |  |  |  |  |  |  |  |  |  |  |  |  |  |  |  |
| 11IGR4.13 | 36 |  |  |  |  |  |  |  |  | 169 | -19---- | ----06-26 |  |  |  |  |  |  |  |
| 11IGR4.12 |  |  | 28 | 120 |  |  |  |  |  |  | -19---- | ----10-22 |  |  |  |  |  |  |  |
| 11IGR4.11 |  |  |  |  |  |  |  |  |  |  |  |  |  |  |  |  |  |  |  |
| 11IGR4.10 |  |  |  |  |  |  |  |  |  |  |  |  |  |  |  |  |  |  |  |
| 11IGR4.9 | 33 | 46 | 26 |  |  |  |  |  |  | 157 | -19-26-- | ----22---- |  |  |  | 0.3 |  |  |  |
| 11IGR4.8 | 28 |  |  | 116 |  | 18 |  | 145 | 8 | 137 | -21-26-- | ----19---- |  |  |  |  |  |  |  |
| 11IGR4.7 | 29 | 35 |  | 127 | 233 | 16 |  | 150 | 0 | 141 | 14-14-21-- | 23-34-17 | 1.6 | 7.7 | 5.5 |  |  | 8.0 |  |
| 11IGR4.6 |  |  | 28 | 118 | 234 | 8 |  | 164 | 7 | – | ----26-- | ----16---- |  |  |  |  | 0.3 |  | 13 |
| 11IGR4.5 | 36 | 45 | 28 | 133 | 248 | 10 | 49 | 162 | -3 | 169 | 18-18-31-25 | 36-22-15 | 1.5 | 6.9 | 5.3 | 0.3 | 0.4 | 6.9 | 11.5 |
| 11IGR4.4 | 31 | 42 | 25 | 114 | 243 | 16 | 63 | 147 | 3 | 149 | -18---- | ----02-29 | 1.6 | 7.8 | 5.6 | 0.2 | 0.7 | 7.8 |  |
| 11IGR4.3 | 31 |  | 26 | 114 | 218 | 15 | 57 | 150 | -4 | 149 | --18-24-19 | ----26-26 | 1.5 | 6.5 | 5.0 | 0.2 | 0.6 | 7.0 | 12 |
| 11IGR4.2 |  |  |  | 114 | 220 | 10 | 46 | 159 | 8 |  | ------17 | ----13-36 |  |  |  |  |  |  |  |
| 11IGR4.1 |  | 37 | 26 | 108 | 217 |  |  |  |  |  | -15-24-- | --------12 |  |  |  |  |  |  |  |
| means | 32 | 41 | 27 | 118 | 230 | 13 | 54 | 154 | 4 | 153 | 16-18-25-20 | 33-19-22 | 1.5 | 7.2 | 5.4 | 0.2 | 0.5 | 7.4 | 12.2 |

|  | FL | FLt | FW | PL | SL | TD | eTW | ANG | FR | H | I-II-III-IV | I^II^III^IV | SL/H | V1 | V2 | (FL-FW)/FW | TD/FW | SL/FL | te |
| --- | --- | --- | --- | --- | --- | --- | --- | --- | --- | --- | --- | --- | --- | --- | --- | --- | --- | --- | --- |
| 15IGR5.5 |  |  |  |  |  |  |  |  |  |  |  |  |  |  |  |  |  |  |  |
| 15IGR5.4 |  |  | 28 | 114 |  | 10 |  | 159 | -6 |  | 22-----24 | 36-21-15 |  |  |  |  | 0.4 |  |  |
| 15IGR5.3 | 32 | 40 | 38 | 105 | 214 | 7 | 40 | 166 | 9 | 153 | 20-18-23-21 | 62-18-21 | 1.4 | 6.1 | 4.8 | -0.2 | 0.2 | 6.7 | 12 |
| 15IGR5.2 | 27 | 43 | 27 | 116 | 219 | 3.5 | 35 | 173 | -3 | 135 | 20-21-27-- | 59-07-11 | 1.6 | 7.3 | 5.3 | 0.0 | 0.1 | 8.0 |  |
| 15IGR5.1 | 35 | 40 | 29 | 107 | 222 |  |  |  |  | 165 | --18-20-- | 57-18-32 | 1.3 | 5.9 | 4.8 | 0.2 |  | 6.3 | 12 |
| means | 31 | 41 | 31 | 111 | 218 | 7 | 37 | 166 | 0 | 151 | 21-19-23-22 | 54-16-20 | 1.4 | 6.5 | 5.0 | 0.0 | 0.2 | 7.0 | 12 |

|  | FL | FLt | FW | PL | SL | TD | eTW | ANG | FR | H | I-II-III-IV | I^II^III^IV | SL/H | V1 | V2 | (FL-FW)/FW | TD/FW | SL/FL | te |
| --- | --- | --- | --- | --- | --- | --- | --- | --- | --- | --- | --- | --- | --- | --- | --- | --- | --- | --- | --- |
| 34IGR10.4 |  | 37 |  | 86 |  |  |  |  |  |  |  |  |  |  |  |  |  |  |  |
| 34IGR10.3 | 28 | 48 | 20 | 150 | 234 | 4 | 38 | 170 | -7 | 137 | 10-16---9 | ----11-17 | 137 | 8.1 | 5.6 | 0.3 | 0.2 | 8.4 | 8 |
| 34IGR10.2 |  | 39 | 26 | 139 | 288 | 4 | 29 | 176 | 1 |  |  | --------32 |  | 11 | 6.9 |  | 0.1 | 10.3 | 12 |
| 34IGR10.1 |  | 35 | 26 |  |  |  |  |  |  |  |  | --------18 |  |  |  |  |  |  | 14 |
| means | 28 | 40 | 24 | 125 | 261 | 4 | 33 | 173 | -3 | 137 | 10-16---9 | ----11-23 | 137 | 9.5 | 6.2 | 0.3 | 0.2 | 9.3 | 11 |
